# The Medical Association of Central New York

**Published:** 1892-07

**Authors:** 


The Medical Association of Central New York held its twenty-fifth annual meeting at Syracuse, Tuesday, May 31, 1892. In point of numbers present and high character of the papers read, it was one of the most successful meetings in its history. Dr. A. A. Hubbell, of Buffalo, N. Y., was its president, and delivered an interesting address on the Abuse of Voluntary Medical Charities. Other papers were read by Drs. Mynter, Ingraham, Stockton, and Putnam, of Buffalo. Papers read by title were those of Drs. Rochester, Thornbury, Crego, and Krauss. Among the Buffalo physicians present were Drs. Hubbell, Ingraham, Stockton, Putnam, Preis, Ring, Krauss, Congdon, Renner, Wende, Jones, VanPeyma, Mynter, and Thornbury.

---

Mrs. Callahan : "Oi want ter git a pair o' shoes fer the little bye."

Clerk : "French kid ?"

Mrs. Callahan (indignantly) : "Indade not. He's me own son, born an' bred in Ameriky."

